# Molecular Characterization of *Enterococcus* Isolates From Different Sources in Estonia Reveals Potential Transmission of Resistance Genes Among Different Reservoirs

**DOI:** 10.3389/fmicb.2021.601490

**Published:** 2021-03-26

**Authors:** Erki Aun, Veljo Kisand, Mailis Laht, Kaidi Telling, Piret Kalmus, Ülo Väli, Age Brauer, Maido Remm, Tanel Tenson

**Affiliations:** ^1^Department of Bioinformatics, Institute of Molecular and Cell Biology, University of Tartu, Tartu, Estonia; ^2^Institute of Technology, University of Tartu, Tartu, Estonia; ^3^Department of Microbiology, Institute of Biomedicine and Translational Medicine, University of Tartu, Tartu, Estonia; ^4^Department of Clinical Veterinary Medicine, Institute of Veterinary Medicine and Animal Sciences, Estonian University of Life Sciences, Tartu, Estonia; ^5^Institute of Agricultural and Environmental Sciences, Estonian University of Life Sciences, Tartu, Estonia

**Keywords:** *E. faecium*, *E. faecalis*, antibiotic resistance, virulence factors, *van* genes, whole-genome sequencing, multi-locus sequence typing, phylogenetic analysis

## Abstract

In this study, we aimed to characterize the population structure, drug resistance mechanisms, and virulence genes of *Enterococcus* isolates in Estonia. Sixty-one *Enterococcus faecalis* and 34 *Enterococcus faecium* isolates were collected between 2012 and 2014 across the country from various sites and sources, including farm animals and poultry (*n* = 53), humans (*n* = 12), environment (*n* = 24), and wild birds (*n* = 44). Clonal relationships of the strains were determined by whole-genome sequencing and analyzed by multi-locus sequence typing. We determined the presence of acquired antimicrobial resistance genes and 23S rRNA mutations, virulence genes, and also the plasmid or chromosomal origin of the genes using dedicated DNA sequence analysis tools available and/or homology search against an *ad hoc* compiled database of relevant sequences. Two *E. faecalis* isolates from human with *vanB* genes were highly resistant to vancomycin. Closely related *E. faecalis* strains were isolated from different host species. This indicates interspecies spread of strains and potential transfer of antibiotic resistance. Genomic context analysis of the resistance genes indicated frequent association with plasmids and mobile genetic elements. Resistance genes are often present in the identical genetic context in strains with diverse origins, suggesting the occurrence of transfer events.

## Introduction

*Enterococcus* is a genus of Gram-positive bacteria, with 67 species, belonging to the lactic acid bacteria from the phylum Firmicutes (Arias and Murray, 2012; Parte, 2014). *Enterococcus* species are non-spore-forming facultative anaerobes tolerant to a wide range of environmental conditions (Bondi et al., 2020), which has enabled them to become widespread in nature especially as a part of the commensal flora of nearly all land animals, including mammals, birds, reptiles, and insects; but, they also occur in soil, plants, and aquatic ecosystems (Sadowy and Luczkiewicz, 2014; Guzman Prieto et al., 2016; Dubin and Pamer, 2017).

In humans, *Enterococcus faecium* and *Enterococcus faecalis* are the two most abundant *Enterococcus* commensals of the gastrointestinal and genitourinary tracts, the oral cavity, the vagina, and the skin. In addition to their commensal role, these two *Enterococcus* species have recently emerged as important human pathogens causing infectious diseases, including endocarditis and bacteremia. Their ability to withstand harsh environmental conditions and their high intrinsic resistance or tolerance to many antimicrobials accompanied by their ability to easily acquire high-level resistance to new antimicrobial agents *via* horizontal gene transfer have enabled them to survive and spread within hospital settings and become one of the leading causes of nosocomial infections (Kayser, 2003; Ørstavik, 2004; Heikens et al., 2007).

Human pathogenic *Enterococcus* poses a threat especially to immunocompromised patients (Kommineni et al., 2016). Due to a variety of intrinsic resistance mechanisms, the therapeutic options for *Enterococcus* infections are limited, and the described ease of acquisition of resistance genes from other bacteria makes the treatment even more challenging. The first-line choices for the treatment of *Enterococcus* infections are β-lactam (for example, ampicillin) and aminoglycoside antibiotics (for example, gentamicin, streptomycin), either separately or in synergetic bactericidal combination (Cetinkaya et al., 2000; Gagetti et al., 2019). Glycopeptide antibiotics like vancomycin (available in United States and Europe) and teicoplanin (available in Europe) are used as the second-line drugs for the treatment of infections caused by β-lactam-resistant *Enterococcus* or in the case of patients with serious β-lactam allergies (Levine, 2006; Arias and Murray, 2012; Richey et al., 2015). However, the vancomycin resistance in *Enterococci* also spreads rapidly, and at present, vancomycin-resistant *Enterococcus* (VRE) can be found all over the world, posing a serious threat to global health (McDonald et al., 1997; WHO, 2017). The last-resort antibiotics against enterococcal infections that cannot be treated with β-lactams or glycopeptides include, for example, oxazolidinones like tedizolid and linezolid, daptomycin, tigecycline, and a synergistic combination of streptogramin A and streptogramin B (Cetinkaya et al., 2000; Arias et al., 2010; Rybak and Roberts, 2015; Ahmed and Baptiste, 2018; Bender et al., 2018; Abbo et al., 2019).

In farm animals, *Enterococcus* infections are uncommon (Aphis., 2014), and they are rarely specifically targeted with antibiotics in these settings. However, as a normal part of their commensal intestinal microbiota, *Enterococcus* spp. are exposed to antibiotics administered to animals to treat or prevent infections caused by other bacteria or given in sub-therapeutic doses to achieve the growth-promoting effects (banned in the EU in 2006 and in the US in 2017 and currently allowed in Brazil and China) (Daniel et al., 2015; Roth et al., 2019; Ibrahim et al., 2020). Therefore, the use of antimicrobials in food animal production has been associated with the development of antimicrobial resistance (AMR) in *Enterococci* (Hayes et al., 2004; Gadde et al., 2018). The antimicrobial-resistant bacteria that have emerged and live in the animal production environment are observed to spread to humans *via* direct or indirect human–animal contact or *via* the consumption of or contact with animal products (Marshall and Levy, 2011; Daniel et al., 2015; Fan and Archbold, 2015). While the *E. faecium* isolates from human samples tend to be of different types than the *E. faecium* isolates from animal samples, the same types of *E. faecalis* isolates have been found in both humans and other animal species. This suggests that the antimicrobial-resistant *Enterococcus* strains may be capable of transmission from animals to humans (Hammerum, 2012). In addition to the possible risk of inter-host transmission, these bacteria harbor a pool of mobile genetic elements and may serve as a reservoir for acquisition of antibiotic resistance genes. Thereby, they could also contribute to the spread of resistance genes by distributing them among Gram-positive bacteria, including the possible transfer of resistance genes from animal-associated *Enterococcus* to human bacteria (Marshall and Levy, 2011; Radhouani et al., 2011).

The resistance genes developed in food animal commensal *Enterococcus* strains can also make their way over time into human pathogenic bacteria after entering into environment and ecosystems *via* manure application as fertilizer or through discharges from the wastewater treatment process (Marshall and Levy, 2011; Daniel et al., 2015). In the environment, the bacterial resistance may be transferred to wild animals living in close association with humans (Blanco et al., 2009; Jardine et al., 2012; Shobrak and Abo-Amer, 2014). Birds of prey, especially migratory raptors, travel long distances through different ecological niches and prey on synanthropic rodents and small birds in urban and rural environments. They potentially become host reservoirs of bacteria of the variety of animals on which they feed and may serve as important indicators of environmental contamination with antimicrobial resistance bacteria of different origins, including those from animal husbandry (Marrow et al., 2009; Radhouani et al., 2011; Stępień-Pyśniak et al., 2018).

In the current work, we isolate *Enterococci* from different host species and environments to characterize the circulating strains in terms of their antimicrobial and virulence profile and map the potential interspecies spread by performing multi-locus sequence typing (MLST) and phylogenetic analysis.

## Materials and Methods

### Collection of Study Materials

The collection of samples was carried out between the years 2012 and 2014. The samples were collected from the environment, wild birds, farm animals, and humans over the territory of Estonia (Supplementary Table S1).

The environmental samples (*n* = 66) were collected during 16 sampling campaigns that covered different seasons. Agriculture-related environmental habitat samples were taken at three farms as follows: (i) slurry and manure, (ii) soil from the fields receiving manure, and (iii) surface water from streams and rivers connected to the fields. City-related environmental samples were collected from a city of 100,000 inhabitants as follows: (i) wastewater treatment plant effluent, (ii) an effluent receiving stream, and (iii) the city environment, including a river inside the city and an artificial outdoor bathing lake connected to the river.

The animal samples were collected from farm animals including poultry, swine, and cattle as well as from wild-living birds, mostly raptors. The farm animal samples included samples from healthy swine and cattle and fecal samples from healthy poultry. The fecal samples from swine and cattle were collected in the course of the annual national *Salmonella* surveillance program carried out in Estonia in 2012–2014. The fecal samples from poultry were collected *post-mortem* in slaughterhouses during the national *Salmonella* surveillance program. All samples were sent to the National Veterinary and Food Laboratory for isolation and identification of *Enterococcus*. The samples from the raptors were collected from the nestlings using cloacal swabbing. Three raptor species foraging in an agricultural landscape were selected for our study: the goshawk *Accipiter gentilis* (feeds mainly on birds in the study area), lesser spotted eagle *Clanga pomarina* (a generalist hunting mostly small mammals), and common buzzard *Buteo* (a generalist with a wide spectrum of diet).

The human samples were collected from two major sources in 2012–2013. Firstly, isolates from the clinical samples of the patients of the largest Estonian hospitals were included. A second source of strains was the fecal samples of healthy volunteers (including pig farmers and dog owners; *n* = 207).

### Isolation Procedures, Vancomycin Resistance Selection, and Testing

The selective cultivation of *Enterococcus* from the environmental samples was carried out according to standardized environmental monitoring methods for the detection and enumeration of major intestinal enterococci (International Organization for Standardization, 1998). The samples were cultured on selective 4-methylumbelliferyl-β-D-glucoside (MUD) microplates (Bio Rad MUD/SF Microplates for *Enterococcus* Test) at 44 ± 0.5°C for 36–72 h. Three positive wells from lowest or second lowest dilution on MUD were selected, and 100 μl per well was used as bacterial suspension for 10-fold dilutions (1:10–1:10–5) with NaCl (0.9%) solution. Then, 50 μl of dilution 1:10 was cultured on the selective VRE agar plates (Oxoid Brilliance VRE Agar) at 37°C for 24–48 h. For a control and total numbering of the *Enterococcus* in the selected well, 50 μl of dilutions 1:10–4 and 1:10–5 was plated on selective Slanetz and Bartley (SB) agar plates for 48 h at 42°C. Three typical colonies from each morphological subset on the VRE plates and one typical colony from SB agar were selected and plated on Luria–Bertani (LB) agar.

The selective cultivation of *Enterococcus* from the fecal samples of farm animals and poultry was carried out by incubating 1 g of feces at 37°C overnight in enrichment broth agar (6.5% NaCl brain heart infusion), and 10 μl of enrichment suspension was spread on Slanetz–Bartley agar and incubated for 48 h at 42°C. Up to four colonies with morphology typical of *Enterococcus* were sub-cultivated on blood agar. Colonies were identified by the following criteria: hemolysis on blood agar, aesculin hydrolysis on Edward’s medium, growth in the presence of tellurite, and the ability to ferment mannitol, sorbitol, arabinose, and raffinose.

The selective cultivation of *Enterococcus* from the cloacal and fecal swab samples of wild birds was carried out by first shacking the swabs on room temperature in 100 ml QSR for 30 min. The solution was filtrated through 45 μm filters, which were then incubated on Slanetz and Bartley agar for 48 h at 42°C. The colonies grown on SB agar were plated with needle on VRE agar plates and incubated at 37°C for 24–48 h. Three typical colonies from each morphological subset on the VRE plates and one typical colony from SB agar were selected and plated on LB agar.

The isolation of *Enterococcus* from human clinical specimens was conducted using standard clinical laboratory methods, which were in accordance with the guidelines of the American Society of Microbiology. The selection of the *Enterococcus* isolates from the fecal samples of human volunteers was carried out by plating the samples on selective medium (BrillianceTM VRE Agar, Oxoid, Basingstoke, United Kingdom). The plates were incubated at 37°C for 24 h, and the negative plates were re-incubated for an additional 24 h. Two colonies per plate with morphology suggestive of *Enterococcus* were selected and confirmed at species level using matrix-assisted laser desorption ionization–time of flight mass spectrometry (Bruker Daltonics, Bremen, Germany).

The minimal inhibitory concentrations of vancomycin for all strains were detected using epsilometer test (Etest, bioMérieux, Marcy l’Etoile, France), and The European Committee on Antimicrobial Susceptibility Testing (EUCAST) breakpoints were used for the interpretation of the results (EUCAST, 2018).

Isolate stocks were made from a single colony (LB plates) of the overnight cultures (LB liquid media). For long-term storage, 15% glycerol stocks of the isolates were made after incubation and stored at −80°C. For DNA extraction and PCR analysis, bacterial pellets were made and stored at—20°C.

### DNA Extraction, Genome Sequencing, and Assembly

DNA was extracted from single bacterial colonies grown on blood agar plates (human isolates) or isolate pellets (grown on LB; all other isolates) using the GuSCN-silica protocol (Boom et al., 1990) modified with bead beating (Telling et al., 2020).

Bacterial genomic DNA was quantified using Qubit^®^ 2.0 Fluorometer (Invitrogen, Grand Island, United States) and 2200 TapeStation (Agilent Technologies, Santa Clara, United States). One nanogram of sample DNA was processed for the sequencing libraries using the Illumina Nextera XT sample preparation kit (Illumina, San Diego, United States) following the manufacturer’s protocols. Libraries were validated by qPCR with Kapa Library Quantification Kit (Kapa Biosystems, Woburn, United States) in order to optimize cluster generation. Ninety-six ssDNA Nextera XT libraries originating from 96 isolates were pooled and sequenced on one high-output lane of HiSeq2500 (Illumina, San Diego, United States), with paired-end, 150 bp reads. Demultiplexing was conducted using CASAVA 1.8.2. (Illumina, San Diego, United States), allowing one mismatch in the index reads. Thereafter, all Illumina reads were assembled *de novo* with the SPAdes genome assembler (ver 3.5.0) using MismatchCorrector (Bankevich et al., 2012).

### Collection of the Reference Genome Set

Sixteen *E. faecalis* and 15 *E. faecium* reference genomes were obtained from RefSeq database (release 90) and included in the analysis for comparison. In selecting the reference genomes for comparison, we prioritized the genomes of strains isolated from nearby countries and/or wild birds, but a set of *Enterococcus* strains from other various sources and distant countries was included as well (for a more detailed description of the included reference strains, see Supplementary Table S2).

### Species Identification, Multi-Locus Sequence Typing, and Population Structure Analysis

The final species identification was conducted from raw sequencing reads of our isolates using StrainSeeker software (Roosaare et al., 2017); this was followed by MLST and phylogenetic analysis to examine the relatedness of our isolates. The MLST type was determined *in silico* using the software MLST^1^ which scans the contig files against traditional PubMLST typing schemes based on the sequence of seven house-keeping genes *gdh*, *gyd*, *pstS*, *gki*, *aroE*, *xpt*, and *yqiL* for *E. faecalis* and *atpA*, *ddl*, *gdh*, *purK*, *gyd*, *pstS*, and *adk* for *E. faecium* (Jolley et al., 2018).

The core-genome alignments of the *E. faecalis* and *E. faecium* isolates were constructed using the ParSNP tool (version 1.2) from the Harvest Suite software for fast multiple alignment of genomic sequences (Treangen et al., 2014). Thereafter, recombinant regions in the core genomes were identified using BRATNextGen software (Marttinen et al., 2012) and masked to create alignments, free of the potential confounding influence of homologous recombination, for phylogenetic analysis. These alignments were used as an input for RaxML-NG software (version 0.7.0 BETA) to reconstruct a maximum likelihood phylogenetic tree using GTR-GAMMA model with four rate categories (Kozlov et al., 2019).

### Determining the Presence of AMR and Virulence Genes, Their Plasmid, or Chromosomal Location and Genomic Context

The presence of acquired and intrinsic resistance genes was determined using ResFinder 3.2 software and ResFinder database as of October 1, 2019 (Zankari et al., 2012). The search was conducted against all AMR classes in the database, with the ResFinder’s minimum coverage cutoff raised from a default of 0.6 to 0.8 and the minimum identity percent cutoff raised from a default of 0.9 to 0.95.

As the *Enterococcus* resistance to last-resort antibiotic linezolid often results from point mutations in polyclonal chromosomal 23S rRNA gene, we used LRE-Finder software tool (Hasman et al., 2019), which is dedicated to detect these mutations and other linezolid resistance-associated genes [*optrA*, *cfr*, *cfr(B)*, and *poxtA*] on the sequencing raw reads of our isolates.

We searched the genomes of our isolates for the presence of the *E. faecalis* and *E. faecium* virulence factors associated in the literature with human infections. The *E. faecalis*-specific virulence genes included in our search were *ace*, *asa1*, *cylA*, *efaA*_*fs*_, *espfs*, *gelE*, hyl*A*, and *hylB* (Singh et al., 1998, 2010; Vankerckhoven et al., 2004; Stępień-Pyśniak et al., 2019; Kiruthiga et al., 2020), and the *E. faecium*-specific virulence factors included in our search were *acm*, *efaA*_*fm*_, *ecbA*, *espfm*, *hylEfm*, *ptsD*, *scm*, *sgrA*, *orf1481*, and IS*16* and four hospital variants of complete pili gene clusters (Freitas et al., 2018; Stępień-Pyśniak et al., 2019). The search was conducted by the alignment of the gene sequences against the genome assemblies using BLASTn (Altschul et al., 1997), with an identity threshold of 95% and gene coverage threshold per hit of 80% (for a more detailed description of the searched virulence genes, see Supplementary Table S3).

The plasmid or chromosomal origin of the detected virulence and antimicrobial resistance factors was determined by combining the results of the PlasmidFinder software tool with default parameters (Carattoli et al., 2014) and BLASTn homology search of the corresponding contigs against the *ad hoc* compiled database of plasmid sequences derived from NCBI RefSeq database as described in Roosaare et al. (2018). The BLAST search was conducted using identity threshold of 70% and plasmid coverage threshold per hit of 10%. The PlasmidFinder results were ignored if the hit for plasmid replicon was found in the complete chromosomal sequence.

The genomic regions containing multiple resistance genes were studied in more detail, and the organization of the genes in these regions was reconstructed by the prediction of gene and corresponding protein sequences in these regions using Prodigal software (Hyatt et al., 2010). The predicted genes were annotated by the comparison of the corresponding protein sequences to available annotated sequences in public databases using BLASTp (Altschul et al., 1997).

### Analysis and Visualization

All analytic scripts were written in Bash^2^ or Python3 (^3^ RRID:SCR_008394) programming languages.

The constructed phylogenetic trees were visualized using Python’s ETE 3 (Environment for Tree Exploration) toolkit (Huerta-Cepas et al., 2016). All the plots were created using Python’s Pandas (McKinney, 2010) and Matplotlib (RRID:SCR_008624) (Hunter, 2007) libraries.

## Results

### Recovery of Enterococcus Isolates

**FIGURE 1:**
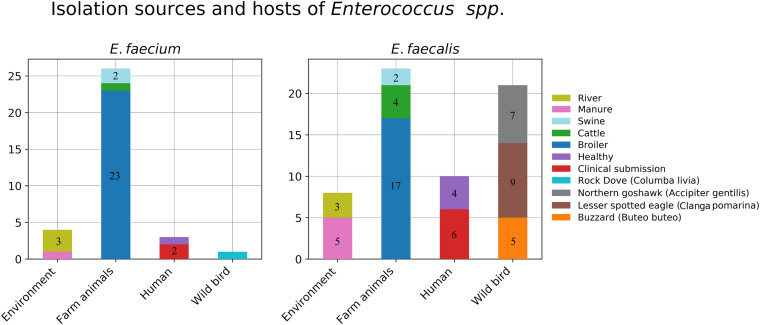
Isolation sources and hosts of the collected *Enterococcus* strains.

In total, 61 *E. faecalis* isolates were recovered from the collected samples and involved in our study. These included eight isolates from environmental sources (river = 3, manure = 5), six isolates from farm animals (*Bos taurus* = 4, *Sus scrofa* = 2), 17 isolates from poultry, 21 isolates from wild raptors (*Buteo buteo* = 5, *A. gentilis* = 7, *C. pomarina* = 9), and nine isolates from human samples (clinical = 6, healthy = 4). The distribution of the isolation sources is plotted in Figure 1.

For *E. faecium*, in total, 34 isolates were recovered from the collected samples, and these included four isolates from environmental sources (river = 3, manure = 1), three isolates from farm animals (*B. taurus* = 1, *S. scrofa* = 2), 23 isolates from poultry, one isolate from free living pigeon *Columba livia*, and three isolates from human samples (clinical = 2, healthy = 1). The distribution of the isolation sources is plotted in Figure 1.

### The MLST Analysis of Enterococcus Isolates

The 61 *E.* faecalis isolates were resolved into 30 sequence types (STs), of which 18 were represented by a single isolate. The most abundant sequence type was ST49, with eight isolates from poultry (*Gallus gallus*), followed by ST936 (six isolates), ST287 (five isolates), and ST4 (four isolates) from wild raptors *A. gentilis*, *Buteo*, and *C. pomarina*, respectively. The *E. faecalis* isolates from hospitalized patients were of sequence types 16, 40, 49, and 774, while three of four colonizing isolates from healthy volunteers were of previously undescribed STs, and one was of sequence type 133. Novel *E. faecalis* sequence types (ST933–ST941 and ST943) were submitted to the PubMLST database (Jolley et al., 2018).

The 34 *E. faecium* isolates were resolved into 24 STs, of which 19 were represented by a single isolate. The most abundant ST was ST258, with six isolates from poultry (*Gallus gallus*). The only human-colonizing *E. faecium* isolate from healthy volunteers was of ST822, and two isolates from hospitalized patients were of ST117. We also discovered six novel *E. faecium* STs, which were submitted to the PubMLST database and assigned to the sequence types ST1634–ST1639.

For *E. faecalis*, ST287 was found in *A. gentilis* and *C. pomarina*, ST4 was found in *G. gallus*, *C. pomarina*, and *B. buteo*, ST49 was found in *G. gallus* and *H. sapiens*, and ST16 was found in *H. sapiens* and *B. taurus* and also in manure. In contrast, all *E. faecium* isolates in our dataset were found within the same host species or environmental origin.

### Detection of Virulence Genes

The virulence genes *ace*, *asa1*, *cylA*, *efaA*, *gelE*, *hylA*, and *hylB* were detected in *E. faecalis* isolates, with the adhesin-like antigen encoding gene *efaA* found to be present in the chromosome of all isolates of this species. It was followed by gelatinase coccolysine gene *gelE*, which was present in 53 (87%), and hyaluronidase gene *hylB* present in 48 (79%) *E. faecalis* isolates. As expected, the aggregation substance encoding gene *asa1* was exclusively found to be located on a plasmid. A plasmid- or chromosome-located cytolysin encoding gene *cylA* was carried by a plasmid in 10 out of 12 (83%) of our samples harboring that gene. Most virulence genes (*asa1* or *ace* and *gelE*, *cylA*, *efaA*, *hylA*, and *hylB*) were detected in six *E. faecalis* strains isolated from wild raptors *A. gentilis* and *C. pomarina*. None of these genes was detected in *E. faecium* isolates.

In *E. faecium* isolates, we found virulence genes IS*16*, *ptsD*, *orf1481*, *efaAfm*, *acm*, *ecbA*, and *scm* and all four pili gene clusters. None of these virulence factors were present in all strains, but the most frequent was collagen adhesin *acm*, which was present in 25 (74%) of our isolates. The pili gene cluster 1 was found in 18 (53%) of our isolates, and it was exclusively located on a plasmid, which is in agreement with previous reports by other investigators. Most virulence genes, IS*16*, *ptsD*, *orf1481*, *acm*, and *ecbA*, and all pili gene clusters were found in the two clinical *E. faecium* isolates HUM-574 and HUM-575. In our strains, the endocarditis-specific antigen *efaAfm* was found only in the plasmid of one environmental isolate ENV-120. The two *efaAfm*-harboring reference strains were also not of human origin.

The surface protein encoding genes *espfs* and *espfm* as well as *E. faecium* virulence genes *hylEfm* and *sgrA* were not found in any of our isolates.

### Antibiotic Susceptibility of Enterococcus Isolates

**FIGURE 2:**
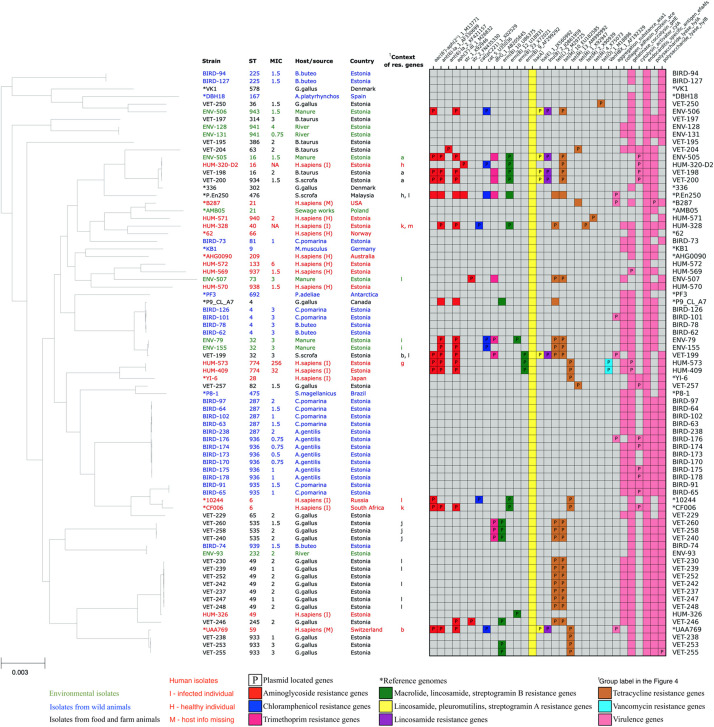
Core-genome phylogenetic tree of our collected 61 *Enterococcus faecium* isolates and 16 reference genomes with the distribution matrix of the antimicrobial resistance and virulence genes detected in their whole-genome sequences.

Two of the *E. faecalis* and none of the E. *faecium* isolates were vancomycin resistant according to EUCAST breakpoints (MIC ≤ 4—sensitive, MIC > 4—resistant) (EUCAST, 2018). These two *E. faecalis* were highly resistant human isolates from sequence type 774. They carried *vanB* gene clusters and showed vancomycin MICs of 256 and 32 mg/L (Figure 2).

### Detection of Antimicrobial Resistance Genes

Genes encoding proteins conferring resistance to aminoglycoside (*aac*, *aph*, *aac*-*aph*, *ant*, and *str*), phenicol (*cat*), trimethoprim (*dfrG*), macrolide (*erm* and *msr*), lincosamide (*erm*, *lnu*, and *lsa*), tetracyclin (*tet*), streptogramin A (*lsa*), streptogramin B (*erm* and *msr*), pleuromutilins (*lsa*), and vancomycin (*van*) antibiotics were found in our isolates. Macrolide and streptogramin B resistance-encoding *msr* genes were found exclusively in *E. faecium* isolates, and vancomycin resistance-encoding *van* genes were found exclusively in *E. faecalis* isolates of human origin, while other major AMR gene classes were detected in isolates of both species and different origins. In our *Enterococcus* isolates, the tetracycline resistance gene variants *tet(L)_2_M29725* and *tet(M)_10_EU182585* (ResFinder database IDs; Zankari et al., 2012) were found together exclusively in the isolates of animal husbandry origin. In contrast, all reference *E. faecium* strains, which harbored these two genes, were strains of human origin. None of the tetracycline resistance genes was detected in the isolates of wild birds. The resistance genes found in this study, together with the antibiotic classes they confer resistance to, and a description of the function of their products are listed in Supplementary Table S4.

#### Resistance Genes in *E. faecalis*

All of our *E. faecalis* isolates showed the presence of species-specific chromosomal gene *lsa(A)*, which is responsible for the intrinsic resistance to lincosamide (clindamycin and lincomycin), pleuromutilin, and streptogramin A (dalfopristin, pristinamycin II, and virginiamycin) antibiotics. Five out of 61 (8%) *E. faecalis* isolates also showed the presence of additional acquired *lsa(E)* gene, probably increasing these isolates’ resistance to the mentioned antibiotics even further. Thirty-one (51%) *E. faecalis* isolates showed the presence of resistance genes against tetracycline antibiotics *tetL*, *tetM*, or *tetO.* Tetracycline resistance genes were often found together with erythromycin-resistant methylase encoding *erm(B)* genes (17 isolates, 28%), aminoglycoside resistance genes (15 isolates, 24%), dihydrofolate reductase encoding *dfrG* genes (nine isolates, 15%), chloramphenicol acetyltransferase encoding *cat* genes (five isolates, 8%), and lincosamide nucleotidyl transferase encoding *lnu* genes (five isolates, 8%). Two human isolates showed the presence of *van* gene cluster conferring resistance to vancomycin, which is one of the most important antibiotics against *Enterococcus*.

In total, acquired resistance genes were found from 32 (53%) of our *E. faecalis* isolates. One isolate had a single *erm(B)* gene, and 11 isolates had acquired resistance genes only for tetracycline. Other 20 isolates possessed resistance genes for more than one class of antibiotics, of which 11 isolates harbored resistance genes from four or more AMR gene classes, potentially conferring them resistance for up to seven different antibiotic classes. These highly multi-resistant isolates of *E. faecalis* originated from humans (five), manure (four), and livestock (three). Although we detected two VRE isolates, we did not detect the resistance genes against ampicillin or linezolid, which are other clinically important antibiotics used to treat *Enterococcus* infections. The distribution of resistance genes in our *E. faecalis* isolates is shown in Figure 2.

#### Resistance Genes in *E. faecium*

All of our *E. faecium* isolates showed the presence of chromosomal *msrC* gene associated with macrolide–streptogramin B resistance and *aac(6’)-Ii_1_L12710* gene associated with the intrinsic low-level resistance to aminoglycosides of this species. Thirteen out of 34 (38%) *E. faecium* isolates also showed the presence of additional acquired aminoglycoside resistance genes, possibly contributing to the higher resistance to aminoglycosides of these strains. The acquired aminoglycoside resistance genes were often found together with erythromycin-resistant methylase encoding *erm* genes (nine isolates, 27%), lincosamide nucleotidyl transferase encoding *lnu* genes (nine isolates, 27%), lincosamide, pleuromutilins, and streptogramin A resistance-associated *lsa* gene (six isolates, 18%), and trimethoprim-resistant dihydrofolate reductase encoding *dfrG* gene (six isolates, 18%). None of the *dfrG*, *erm*, *lnu*, or *lsa* genes was found in isolates without acquired aminoglycoside resistance genes. In addition, four (12%) isolates showed the presence of chloramphenicol acetyltransferase-encoding *cat* genes, and 10 isolates (29%) showed the presence of tetracycline resistance genes *tetL* or *tetM.*

**FIGURE 3:**
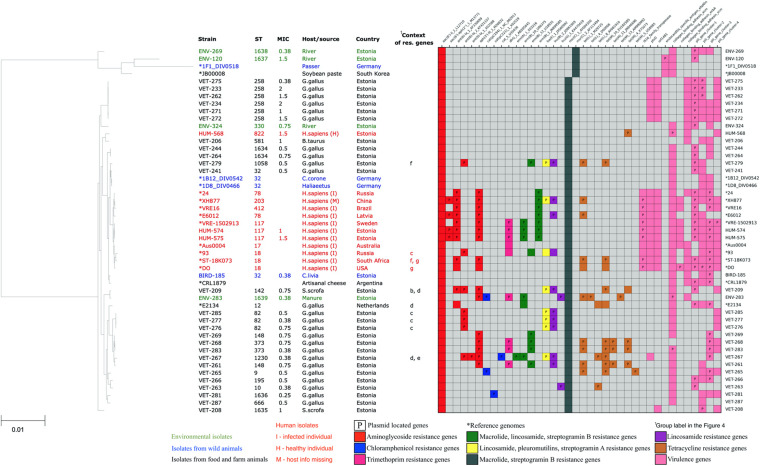
Core-genome phylogenetic tree of our collected 34 *Enterococcus faecium* isolates and 16 reference genomes with the distribution matrix of the antimicrobial resistance and virulence genes detected in their whole-genome sequences.

In total, acquired resistance genes were found in 17 (50%) of our *E. faecium* isolates. Fifteen of our isolates possessed acquired resistance genes for more than one class of antibiotics, with nine isolates possessing acquired resistance genes from four or more AMR gene classes, potentially conferring them resistance for up to seven different antibiotic classes. These highly multi-resistant isolates of *E. faecium* originated from poultry (five), livestock (one), manure (one), and humans (two). However, we did not detect the resistance genes or mutations against vancomycin, ampicillin, or linezolid, which are the clinically most important antibiotics against *Enterococcus* infections. The distribution of resistance genes in our *E. faecium* isolates is shown in Figure 3.

### Genomic Context of the Resistance Genes

**FIGURE 4:**
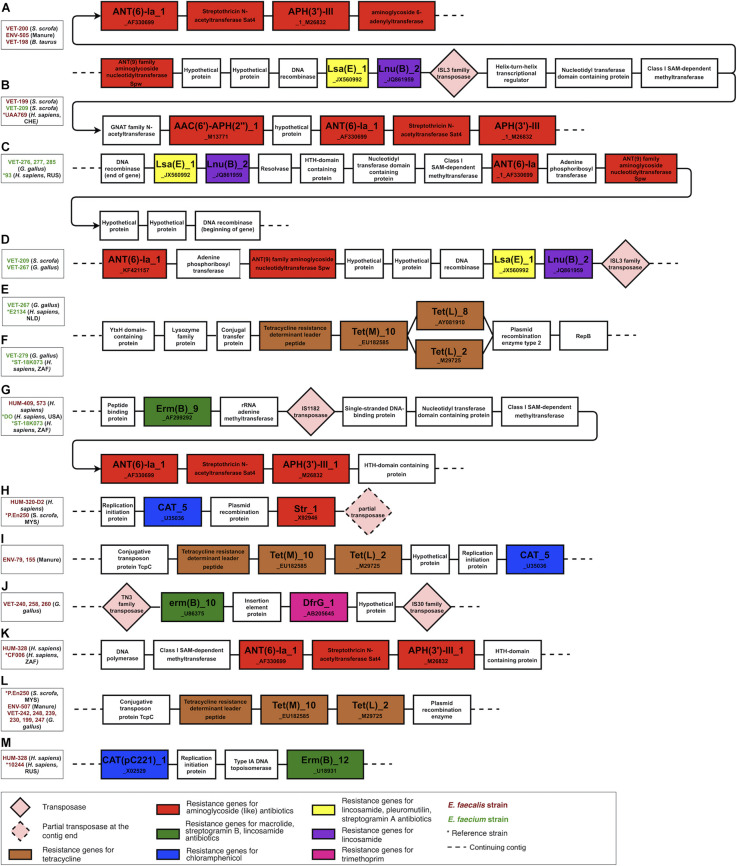
The genomic context of the resistance genes in the antibiotic resistance islands. Most of the clustered resistance genes were found in the same genetic context in strains of different origins and were flanked by mobile genetic elements and plasmid-related genes. The figure illustrates only the context and is not scaled to the actual length of the genes and intergenic sequences. **(A–D)** a *lsa(E)*, *lnu(B)* and aminoglycoside resistance genes harboring genomic islands found in husbandry and human related strains, **(E,F,L)** a tetracycline resistance gene cluster variants found in human and husbandry related strains, **(G)** a *erm(B)* and aminoglycoside resistance genes harboring genomic in human related strains, **(H)** a *cat_5* and *str_1* genes harboring genomic island in human and husbandry related strains, **(I)** a tetracycline resistance cluster and *cat_5* gene harboring genomic island found in husbandry related strains, **(J)** a *erm(B)* and *dfr(G)* genes harboring genomic island found in poultry related strains, **(K)** an aminoglycoside resistance genes harboring genomic island found in human related strains, **(M)** *cat(pc221)_1* and *erm(B)* genes harbouring genomic island found in human related strains.

The genomic contexts of the resistance genes in the antibiotic resistance islands were reconstructed and schematically illustrated in Figure 4. This analysis confirmed the plasmid origin of these genes, as many of them were flanked by plasmid-related genes, for example, plasmid recombination enzyme encoding genes next to tetracycline resistance genes (regions e, f, and l in Figure 4) and between chloramphenicol and streptomycin resistance genes (region h in Figure 4). The studied resistance genes were also often flanked by transposon-specific genes. We found the transposase genes in regions a, b, d, g, h, and j and the transposon protein TcpC-encoding genes in regions i and l in Figure 4. The resistance genes were found in the identical genetic context in the strains of different isolation sources or geographical origins (comparison strains). Furthermore, some resistance genes were found in the same genetic context in *E. faecium* and *E. faecalis* isolates. These findings suggest that plasmids and transposons play an important role in the dissemination of resistance genes from and to *Enterococcus.* This genetic context analysis also reveals that the tetracycline resistance genes *tet(L)_2_M29725* and *tet(M)_10_EU182585*, which in our isolated strains are exclusively associated with animal husbandry origin, are adjacent genes included into a leader peptide-controlled tetracycline resistance cluster.

The genomic context analysis also revealed that the aminoglycoside resistance genes *ant(6)-Ia_1_AF330699*, *aph(3’)-III_1_M26832*, lincosamide resistance gene *lnu(B)_2_JQ861959*, and lincosamide, pleuromutilins, and streptogramin A resistance gene *lsa(E)_1_JX560992* are located in close proximity in a region named *lsa(E)*-carrying multiresistance gene cluster (Si et al., 2015). This multiresistance gene cluster was present in three strains, which were isolated from different animal husbandry-related sources (manure, *B. taurus*, *S. scrofa*) but were phylogenetically similar and clustered into one branch in the phylogenetic tree. The fourth strain from this branch was isolated from a tracheal aspirate of a person with pneumonia, and this strain was missing this multiresistance gene cluster.

## Discussion

Both *E. faecalis* and *E. faecium* are known to colonize different animal species. In our dataset, we have *E. faecalis* sequence types colonizing different species. ST287 was shared between different raptor species. ST4 was shared between broiler and wild bird samples (*C. pomarina* and *B. buteo*). ST16 was shared by humans and cattle, and ST49 was isolated from human and broiler samples, which has been also described by other researchers (Braga et al., 2018). In contrast, all *E. faecium* strains in our dataset were found within the same host species or environmental origin. This is in accordance with previous findings that *E. faecalis* strains of the same sequence type can be found in humans as well as in other animal species, while *E. faecium* strains tend to be more host specific (Hammerum, 2012).

Two of our *E. faecium* strains isolated from hospitalized patients had the multi-resistance sequence type 117 associated with nosocomial outbreaks in multiple European countries (Tedim et al., 2017). Although multi-resistant, we did not find our ST117 strains to possess vancomycin resistance genes as confirmed by vancomycin susceptibility testing, which is in contrast to many other reports (Papagiannitsis et al., 2017; Falgenhauer et al., 2019; Olearo et al., 2021). Our only human-colonizing *E.* faecium strain isolated from healthy patients was of sequence type 822, and it did not show the presence of any acquired resistance genes, although strains of the same sequence type are described as multi-resistant, including resistance to vancomycin, by other researchers (Castro-Nallar et al., 2017; Zangue, 2017).

For *E. faecalis*, we had two vancomycin-resistant isolates obtained in 2013 from the wound discharges of hospitalized patients. These isolates belonged to ST774, which is assigned to uropathogenic strains by other researchers (Elena Aleksandrovna et al., 2019; Strateva et al., 2019). Strateva et al. describe in their article the *vanA* gene cluster carrying vancomycin-resistant *E. faecalis* strains of ST774 in Bulgaria in 2015. In contrast, our strains of ST774 were vancomycin resistant due to the presence of *vanB* genes. Other *E. faecalis* strains from hospitalized patients belonged to the sequence types ST16, ST40, and ST49, which are described as widespread STs obtained from different sources and conditions, including human clinical infections, healthy volunteers as well as animals and environment (Ruiz-Garbajosa et al., 2006; Zischka et al., 2015; Braga et al., 2018). ST133 was the only previously described ST of our *E. faecalis* isolates from healthy volunteers. In this isolate (HUM-572), we detected no acquired resistance genes and only one of the searched virulence gene. The strain belonging to ST133 has also been shown as a gut colonizer of healthy individuals in another study (Moles et al., 2015).

It has been described that the virulence of *Enterococcus* is associated with the presence of certain virulence genes. We have analyzed the genomes for eight virulence genes described mainly in *E. faecalis* and 14 virulence genes or gene clusters specific to *E. faecium*. In *E. faecalis*, we did not observe a clear enrichment of the virulence genes in human-derived strains. We instead found the highest number of virulence genes in strains isolated from wild raptors *A. gentilis* and *C. pomarina.* In contrast, in *E. faecium*, we observed evident enrichment of virulence genes in human clinical isolates as compared to human commensal or non-human isolates. However, all of our isolates carried at least one virulence gene, with *E. faecalis asa1* carried only by two raptor- and one swine-derived strain and *E. faecium efaAfm* carried only by one environmental isolate. With that in mind, we can only speculate if these non-human virulence gene-carrying strains can contribute to the virulence of human pathogenic strains when coming into contact with them or if they are able to cause diseases themselves when transferred to humans, making wild and domesticated birds potential reservoirs for zoonotic outbreaks.

Several antibiotic resistance genes were detected. As described previously, *lsa(A)*, causing resistance to lincosamides, pleuromutilines, and streptogramin A, was present in all strains of *E. faecalis*. All *E. faecium* isolates had a chromosomal *msrC* gene associated with macrolide–streptogramin B resistance and *aac(6’)-Ii_1_L12710* gene associated with the intrinsic low-level resistance to aminoglycosides. These genes contribute to the intrinsic antibiotic resistance of *Enterococcus*. Moreover, 53% of *E. faecalis* strains and 50% of *E. faecium* strains have genes for acquired antibiotic resistance. Most strains with acquired resistance genes contain multiple genes predicted to give resistance to several antibiotics. As the acquired resistance genes are often located on transposons and/or plasmids, it is expected that the resistance can be transferred between different strains. This is expected to contribute to the constantly increasing resistance levels in *Enterococcus* (Manson et al., 2010; Palmer et al., 2010).

The expected transmission of resistance genes by mobile genetic elements is in accordance with our finding that the resistance genes are often present in the identical genetic context in strains with diverse origins. Furthermore, most of the detected resistance genes were found in strains isolated from different hosts or environmental sources, with the exception of the tetracycline resistance gene cluster with *tet(M)* and *tet(L)* genes, which was found exclusively in our strains of animal husbandry origin. Nevertheless, the presence of this tetracycline gene cluster in human comparison strains from other countries indicates a potential for transmission of this gene cluster to human strains, where it could pose additional health risks related to antimicrobial resistance. The absence of *lsa(E)*-carrying multi-resistance gene cluster (Figure 4) in the strain of human origin but its presence in other phylogenetically similar strains of animal husbandry origin suggests a possible cluster loss event in this human-related strain isolated from the tracheal aspirate of a person with pneumonia. None of the strains isolated from wild birds harbored acquired resistance genes, which indicates that the environmental contamination with antibiotics and AMR genes is low in Estonia and that neither wild raptors nor their prey is coming into contact with antimicrobials or bacteria capable of transferring resistance genes.

Vancomycin is one of the critical antibiotics against infections caused by multi-resistant *Enterococcus*. This resistance is caused by *van* genes. Complete *vanB* gene clusters were found from two (3%) of *E. faecalis* strains. As expected, these two strains containing the *vanB* gene cluster were resistant to vancomycin. During the sample collection period, the prevalence of VRE in Estonia was low. As the resistance levels are increasing globally, continuous monitoring and research is needed for mapping the spread and potentially designing containment measures.

## Conclusion

Our study has shown the widespread prevalence of acquired resistance genes among *Enterococcus* strains exposed to anthropogenic antibiotic pressure. Additionally, the study has also shown that *E. faecalis* strains colonizing different farm animal species and humans could be closely related and contain many potentially mobile antibiotic resistance genes that can contribute to the spread of resistance among different reservoirs. However, no mobile resistance genes were found in *Enterococcus* from free-living birds, which suggests that areas with lower contamination with antibiotics or AMR genes still exist and that the spread of antibiotic resistance to wildlife can be prevented or postponed. The restricted use of antibiotics in animal husbandry and the elimination of potential resistance transmission routes are essential to maintain the currently low prevalence of resistance genes in wildlife in Estonia.

## Data Availability Statement

The datasets presented in this study can be found in online repositories. The names of the repository/repositories and accession number(s) can be found below: https://www.ncbi.nlm.nih.gov/bioproject/PRJNA630475, PRJNA630475.

## Ethics Statement

The studies involving human participants were reviewed and approved by the Research Ethics Committee of the University of Tartu. Written informed consent to participate in this study was provided by the participants and/or their legal guardian/next of kin for minors. For the animal studies, neither written informed consents from the owners nor the ethical review and approval were obtained or required, as collection of fecal samples is not considered an animal experiment according to Estonian law. Also, the analyzed animal fecal samples were collected earlier in the course of the national *Salmonella* surveillance programme and were already present in laboratory.

## Author Contributions

EA contributed to formal analysis, manuscript writing, and preparation. VK contributed to data curation, resources, manuscript writing, and review. ML and PK contributed to resources, manuscript writing, and review. KT contributed to resources and manuscript review. ÜV contributed to resources and manuscript review. AB contributed to supervision and manuscript review. MR contributed to funding acquisition, project administration, supervision, and manuscript review. TT contributed to funding acquisition, project administration, data curation, resources, manuscript writing, and review. All authors contributed to the article and approved the submitted version.

## Conflict of Interest

The authors declare that the research was conducted in the absence of any commercial or financial relationships that could be construed as a potential conflict of interest.

## Footnotes

1 https://github.com/tseemann/mlst

2 https://www.gnu.org/software/bash/

3 https://www.python.org/

## Abbreviations

AMR: antimicrobial resistance
MLST: multi-locus sequence type
ST: sequence type
VRE: vancomycin-resistant *Enterococcus.*
