# Supplementary material for: Molecular Characterization of Enterococcus Isolates From Different Sources in Estonia Reveals Potential Transmission of Resistance Genes Among Different Reservoirs
Source: Front Microbiol. 2021 Mar 26;12:601490. doi: 10.3389/fmicb.2021.601490 (PMC8032980; doi:10.3389/fmicb.2021.601490)
Supplement: Supplementary file 2 [file Table_2.DOCX]

**Table 1:** Reference strains included in our study for comparison

| **Species** | **Strain name** | **Host and source** | **Isolation country** | **Collection date** | **References** |
| --- | --- | --- | --- | --- | --- |
| *E. faecium* | 1B12_DIV0542 | Carrion crow (*Corvus corone*); feces | Germany | - | BioProject Accession [PRJNA313452](http://www.ncbi.nlm.nih.gov/bioproject/?term=PRJNA313452);  BioSample Accession [SAMN04634031](http://www.ncbi.nlm.nih.gov/biosample/SAMN04634031);  Assembly Accession [GCA_002140435.1](http://www.ncbi.nlm.nih.gov/assembly/GCA_002140435.1);  GenBank Accessions [NGLW00000000](http://www.ncbi.nlm.nih.gov/nuccore/NGLW00000000); |
| *E. faecium* | 1D8_DIV0466 | Sea eagle (*Haliaeetus*), feces | Germany, Berlin | - | BioProject Accession [PRJNA313452](http://www.ncbi.nlm.nih.gov/bioproject/?term=PRJNA313452);  BioSample Accession [SAMN04634012](http://www.ncbi.nlm.nih.gov/biosample/SAMN04634012);  Assembly Accession [GCA_002141135.1](http://www.ncbi.nlm.nih.gov/assembly/GCA_002141135.1);  GenBank Accessions [NGLT00000000](http://www.ncbi.nlm.nih.gov/nuccore/NGLT00000000); |
| *E. faecium* | 1F1_DIV0518 | Sparrow (*Passer*), feces | Germany, Berlin | - | BioProject Accession [PRJNA313452](http://www.ncbi.nlm.nih.gov/bioproject/?term=PRJNA313452);  BioSample Accession [SAMN04634013](http://www.ncbi.nlm.nih.gov/biosample/SAMN04634013);  Assembly Accession [GCA_002141175.1](http://www.ncbi.nlm.nih.gov/assembly/GCA_002141175.1);  GenBank Accessions [NGLS00000000](http://www.ncbi.nlm.nih.gov/nuccore/NGLS00000000); |
| *E. faecium* | 24 | Human (*Homo sapiens*), feces | Russia, St. Petersburg | Nov-2012 | BioProject Accession [PRJNA412735](http://www.ncbi.nlm.nih.gov/bioproject/?term=PRJNA412735);  BioSample Accession [SAMN07725774](http://www.ncbi.nlm.nih.gov/biosample/SAMN07725774);  Assembly Accession [GCA_002630975.1](http://www.ncbi.nlm.nih.gov/assembly/GCA_002630975.1);  GenBank Accessions [PDMA01000000](http://www.ncbi.nlm.nih.gov/nuccore/PDMA01000000); |
| *E. faecium* | 93 | Human (*Homo sapiens*), skin | Russia, St. Petersburg | May-2013 | BioProject Accession [PRJNA412735](http://www.ncbi.nlm.nih.gov/bioproject/?term=PRJNA412735);  BioSample Accession [SAMN07725778](http://www.ncbi.nlm.nih.gov/biosample/SAMN07725778);  Assembly Accession [GCA_002562875.1](http://www.ncbi.nlm.nih.gov/assembly/GCA_002562875.1);  GenBank Accessions [PDFN01000000](http://www.ncbi.nlm.nih.gov/nuccore/PDFN01000000); |
| *E. faecium* | Aus0004 | Human (*Homo sapiens*), bloodstream | Australia, Melbourne | 1998 | BioProject Accession [PRJNA86649](http://www.ncbi.nlm.nih.gov/bioproject/?term=PRJNA86649);  BioSample Accession [SAMN02604218](http://www.ncbi.nlm.nih.gov/biosample/SAMN02604218);  Assembly Accession [GCA_000250945.1](http://www.ncbi.nlm.nih.gov/assembly/GCA_000250945.1);  GenBank Accessions [CP003351.1, CP003352.1, CP003353.1, CP003354.1;](http://www.ncbi.nlm.nih.gov/nuccore/CP003351.1,CP003352.1,CP003353.1,CP003354.1) |
| *E. faecium* | CRL1879 | Artisanal cheese | Argentina, Tucuman | 2011 | BioProject Accession [PRJNA191091](http://www.ncbi.nlm.nih.gov/bioproject/?term=PRJNA191091);  BioSample Accession [SAMN02469460](http://www.ncbi.nlm.nih.gov/biosample/SAMN02469460);  Assembly Accession [GCA_000406365.1](http://www.ncbi.nlm.nih.gov/assembly/GCA_000406365.1);  GenBank Accessions [AOUK00000000](http://www.ncbi.nlm.nih.gov/nuccore/AOUK00000000); |
| *E. faecium* | DO (TX0016) | Human (*Homo sapiens*), bloodstream | United States, Texas | 1992 | BioProject Accession [PRJNA30627](http://www.ncbi.nlm.nih.gov/bioproject/?term=PRJNA30627);  BioSample Accession [SAMN00002237](http://www.ncbi.nlm.nih.gov/biosample/SAMN00002237);  Assembly Accession [GCA_000174395.2](http://www.ncbi.nlm.nih.gov/assembly/GCA_000174395.2);  GenBank Accessions [ACIY01000000](http://www.ncbi.nlm.nih.gov/nuccore/ACIY01000000); |
| *E. faecium* | E2134 | Chicken (*Gallus gallus*) | Netherlands | 2004 | BioProject Accession [PRJNA82489](http://www.ncbi.nlm.nih.gov/bioproject/?term=PRJNA82489);  BioSample Accession [SAMN00779834](http://www.ncbi.nlm.nih.gov/biosample/SAMN00779834);  Assembly Accession [GCA_000322185.1](http://www.ncbi.nlm.nih.gov/assembly/GCA_000322185.1);  GenBank Accessions [AHXU00000000](http://www.ncbi.nlm.nih.gov/nuccore/AHXU00000000); |
| *E. faecium* | E6012 | Human (*Homo sapiens*) | Latvia | 2010 | BioSample Accession [SAMN00779851](http://www.ncbi.nlm.nih.gov/biosample/SAMN00779851);  Assembly Accession [GCA_000322445.1](http://www.ncbi.nlm.nih.gov/assembly/GCA_000322445.1);  GenBank Accessions [AHYK00000000](http://www.ncbi.nlm.nih.gov/nuccore/AHYK00000000); |
| *E. faecium* | ST-18:K073 | Human (*Homo sapiens*), urine | South Africa, Pretoria | 17-May-2013 | BioProject Accession [PRJNA355910](http://www.ncbi.nlm.nih.gov/bioproject/?term=PRJNA355910);  BioSample Accession [SAMN06106844](http://www.ncbi.nlm.nih.gov/biosample/SAMN06106844);  Assembly Accession [GCA_002416685.1](http://www.ncbi.nlm.nih.gov/assembly/GCA_002416685.1);  GenBank Accessions [NXIX01000000](http://www.ncbi.nlm.nih.gov/nuccore/NXIX01000000); |
| *E. faecium* | JB00008 | Fermented soybean paste | South Korea | 2015-01-19 | BioProject Accession [PRJNA395273](http://www.ncbi.nlm.nih.gov/bioproject/?term=PRJNA395273);  BioSample Accession [SAMN07374792](http://www.ncbi.nlm.nih.gov/biosample/SAMN07374792);  GenBank Accessions [NNSU01000000](http://www.ncbi.nlm.nih.gov/nuccore/NNSU01000000); |
| *E. faecium* | VRE-1502913 | Human (*Homo sapiens*), feces | Sweden, Uppsala | 2015 | BioProject Accession [PRJNA301929](http://www.ncbi.nlm.nih.gov/bioproject/?term=PRJNA301929);  BioSample Accession [SAMN04270972](http://www.ncbi.nlm.nih.gov/biosample/SAMN04270972);  Assembly Accession [GCF_001563065.1](http://www.ncbi.nlm.nih.gov/assembly/GCF_001563065.1);  GenBank Accessions [LNOV00000000](http://www.ncbi.nlm.nih.gov/nuccore/LNOV00000000); |
| *E. faecium* | VRE 16 | Human (*Homo sapiens*), perianal swab | Lebanon | 2018 | BioProject Accession [PRJNA605642](http://www.ncbi.nlm.nih.gov/bioproject/?term=PRJNA605642);  BioSample Accession [SAMN14073388](http://www.ncbi.nlm.nih.gov/biosample/SAMN14073388);  GenBank Accessions [JAALOL010000100, JAALOL010000101, JAALOL010000102, JAALOL010000103, JAALOL010000104, JAALOL010000105, JAALOL010000106;](http://www.ncbi.nlm.nih.gov/nuccore/JAALOL010000100,JAALOL010000101,JAALOL010000102,JAALOL010000103,JAALOL010000104,JAALOL010000105,JAALOL010000106) |
| *E. faecium* | XH877 | Human (*Homo sapiens*), feces | China, Zhejiang | 2012 | BioProject Accession [PRJNA321783](http://www.ncbi.nlm.nih.gov/bioproject/?term=PRJNA321783);  BioSample Accession [SAMN05003824](http://www.ncbi.nlm.nih.gov/biosample/SAMN05003824);  Assembly Accession [GCA_001676845.1](http://www.ncbi.nlm.nih.gov/assembly/GCA_001676845.1);  GenBank Accessions [LXWZ00000000](http://www.ncbi.nlm.nih.gov/nuccore/LXWZ00000000); |
| *E. faecalis* | 10244 | Human (*Homo sapiens*), bloodstream | Russia, Moscow | Aug-2010 | BioProject Accession [PRJNA198772](http://www.ncbi.nlm.nih.gov/bioproject/?term=PRJNA198772);  BioSample Accession [SAMN02470829](http://www.ncbi.nlm.nih.gov/biosample/SAMN02470829);  Assembly Accession [GCA_000438765.1](http://www.ncbi.nlm.nih.gov/assembly/GCA_000438765.1);  GenBank Accessions [ASWX00000000](http://www.ncbi.nlm.nih.gov/nuccore/ASWX00000000); |
| *E. faecalis* | 336 | Chicken (*Gallus gallus*) | Denmark | 23-Oct-2013 | BioProject Accession [PRJNA423145](http://www.ncbi.nlm.nih.gov/bioproject/?term=PRJNA423145);  BioSample Accession [SAMN08211909](http://www.ncbi.nlm.nih.gov/biosample/SAMN08211909);  Assembly Accession [GCA_002848255.1](http://www.ncbi.nlm.nih.gov/assembly/GCA_002848255.1);  GenBank Accessions [PKGH01000000](http://www.ncbi.nlm.nih.gov/nuccore/PKGH01000000); |
| *E. faecalis* | 62 | Human (*Homo sapiens*) | Norway | 2006 | BioProject Accession [PRJNA61185](http://www.ncbi.nlm.nih.gov/bioproject/?term=PRJNA61185);  BioSample Accession [SAMN02603509](http://www.ncbi.nlm.nih.gov/biosample/SAMN02603509);  Assembly Accession [GCA_000211255.1](http://www.ncbi.nlm.nih.gov/assembly/GCA_000211255.1);  GenBank Accessions [CP002491, CP002492, CP002495, CP002493, CP002494;](http://www.ncbi.nlm.nih.gov/nuccore/CP002491,CP002492,CP002495,CP002493,CP002494) |
| *E. faecalis* | AHG0090 | Human (*Homo sapiens*), feces | Australia, Brisbane | 2014 | BioProject Accession [PRJNA413279](http://www.ncbi.nlm.nih.gov/bioproject/?term=PRJNA413279);  BioSample Accession [SAMN07738885](http://www.ncbi.nlm.nih.gov/biosample/SAMN07738885);  Assembly Accession [GCA_003047245.1](http://www.ncbi.nlm.nih.gov/assembly/GCA_003047245.1);  GenBank Accessions [PDUN01000000](http://www.ncbi.nlm.nih.gov/nuccore/PDUN01000000); |
| *E. faecalis* | AMB05 | Water purification plant | Poland, Orzysz | Aug-2013 | BioProject Accession [PRJNA324367](http://www.ncbi.nlm.nih.gov/bioproject/?term=PRJNA324367);  BioSample Accession [SAMN05200884](http://www.ncbi.nlm.nih.gov/biosample/SAMN05200884);  Assembly Accession [GCF_001931845.1](http://www.ncbi.nlm.nih.gov/assembly/GCF_001931845.1);  GenBank Accessions [CP015998](http://www.ncbi.nlm.nih.gov/nuccore/CP015998); |
| *E. faecalis* | B287 | Human (*Homo sapiens*) | United States, Wisconsin | 22-Dec-1984 | BioProject Accession [PRJNA217004](http://www.ncbi.nlm.nih.gov/bioproject/?term=PRJNA217004);  BioSample Accession [SAMN02333877](http://www.ncbi.nlm.nih.gov/biosample/SAMN02333877);  Assembly Accession [GCA_000519445.1](http://www.ncbi.nlm.nih.gov/assembly/GCA_000519445.1);  GenBank Accessions [JAHG00000000](http://www.ncbi.nlm.nih.gov/nuccore/JAHG00000000); |
| *E. faecalis* | CF006 | Human (*Homo sapiens*), urine | South Afria, Pretoria | 26-Jul-2013 | BioProject Accession [PRJNA355910](http://www.ncbi.nlm.nih.gov/bioproject/?term=PRJNA355910);  BioSample Accession [SAMN06106879](http://www.ncbi.nlm.nih.gov/biosample/SAMN06106879);  Assembly Accession [GCA_002417225.1](http://www.ncbi.nlm.nih.gov/assembly/GCA_002417225.1);  GenBank Accessions [NXKG01000000](http://www.ncbi.nlm.nih.gov/nuccore/NXKG01000000); |
| *E. faecalis* | DBH18 | Mallard duck (*Anas platyrhynchos*), intestinal content and carcasses | Spain, Valencia | 12-Oct-2005 | BioProject Accession [PRJNA310065](http://www.ncbi.nlm.nih.gov/bioproject/?term=PRJNA310065);  BioSample Accession [SAMN04445878](http://www.ncbi.nlm.nih.gov/biosample/SAMN04445878);  Assembly Accession [GCF_001563075.1](http://www.ncbi.nlm.nih.gov/assembly/GCF_001563075.1);  GenBank Accessions [LSFS00000000](http://www.ncbi.nlm.nih.gov/nuccore/LSFS00000000); |
| *E. faecalis* | KB1 | Mouse (*Mus musculus*), intestinal content | Germany, Munich | 28-Oct-2011 | BioProject Accession [PRJNA289613](http://www.ncbi.nlm.nih.gov/bioproject/?term=PRJNA289613);  BioSample Accession [SAMN03856105](http://www.ncbi.nlm.nih.gov/biosample/SAMN03856105);  Assembly Accession [GCA_002221625.2](http://www.ncbi.nlm.nih.gov/assembly/GCA_002221625.2);  GenBank Accessions [CP022712](http://www.ncbi.nlm.nih.gov/nuccore/CP022712); |
| *E. faecalis* | P8-1 | Magellanic penguin (*Spheniscus magellanicus*), feces | Brazil, Cidreira | 2013-10-14 | BioProject Accession [PRJNA296376](http://www.ncbi.nlm.nih.gov/bioproject/?term=PRJNA296376);  BioSample Accession [SAMN04100212](http://www.ncbi.nlm.nih.gov/biosample/SAMN04100212);  Assembly Accession [GCF_001400055.1](http://www.ncbi.nlm.nih.gov/assembly/GCF_001400055.1);  GenBank Accessions [LKGR01000000](http://www.ncbi.nlm.nih.gov/nuccore/LKGR01000000); |
| *E. faecalis* | P9_CL_A7 | Chicken (*Gallus gallus*), cloaca | Canada, Briti Columbia | 2005 | BioProject Accession [PRJNA273513](http://www.ncbi.nlm.nih.gov/bioproject/?term=PRJNA273513);  BioSample Accession [SAMN10272814](http://www.ncbi.nlm.nih.gov/biosample/SAMN10272814);  Assembly Accession [GCA_003797725.1](http://www.ncbi.nlm.nih.gov/assembly/GCA_003797725.1);  GenBank Accessions [RKPW01000000](http://www.ncbi.nlm.nih.gov/nuccore/RKPW01000000); |
| *E. faecalis* | P.En250 | Swine (*Sus scrofa*), feces | Malaysia | 2012 | BioProject Accession [PRJNA343058](http://www.ncbi.nlm.nih.gov/bioproject/?term=PRJNA343058);  BioSample Accession [SAMN05772844](http://www.ncbi.nlm.nih.gov/biosample/SAMN05772844);  Assembly Accession [GCF_002009545.1](http://www.ncbi.nlm.nih.gov/assembly/GCF_002009545.1);  GenBank Accessions [MJBZ00000000](http://www.ncbi.nlm.nih.gov/nuccore/MJBZ00000000); |
| *E. faecalis* | PF3 | Adelie penguin (*Pygoscelis adeliae*), feces | Antarctica, Warriner Island | 2010 | BioProject Accession [PRJNA223434](http://www.ncbi.nlm.nih.gov/bioproject/?term=PRJNA223434);  BioSample Accession [SAMN02380586](http://www.ncbi.nlm.nih.gov/biosample/SAMN02380586);  Assembly Accession [GCA_000505585.1](http://www.ncbi.nlm.nih.gov/assembly/GCA_000505585.1);  GenBank Accessions [AZIA00000000](http://www.ncbi.nlm.nih.gov/nuccore/AZIA00000000); |
| *E. faecalis* | UAA769 | Human (*Homo sapiens*) | Switzerland, Geneve | 1996 | BioProject Accession [PRJNA88239](http://www.ncbi.nlm.nih.gov/bioproject/?term=PRJNA88239);  BioSample Accession [SAMN00808933](http://www.ncbi.nlm.nih.gov/biosample/SAMN00808933);  Assembly Accession [GCA_000391565.1](http://www.ncbi.nlm.nih.gov/assembly/GCA_000391565.1);  GenBank Accessions [AISC00000000](http://www.ncbi.nlm.nih.gov/nuccore/AISC00000000); |
| *E. faecalis* | VK1 | Chicken (*Gallus gallus*) | Denmark | 17-Sep-2013 | BioProject Accession [PRJNA423151](http://www.ncbi.nlm.nih.gov/bioproject/?term=PRJNA423151);  BioSample Accession [SAMN08211924](http://www.ncbi.nlm.nih.gov/biosample/SAMN08211924);  Assembly Accession [GCA_002848295.1](http://www.ncbi.nlm.nih.gov/assembly/GCA_002848295.1);  GenBank Accessions [PKGK01000000](http://www.ncbi.nlm.nih.gov/nuccore/PKGK01000000); |
