# Supplementary material for: Molecular Characterization of Enterococcus Isolates From Different Sources in Estonia Reveals Potential Transmission of Resistance Genes Among Different Reservoirs
Source: Front Microbiol. 2021 Mar 26;12:601490. doi: 10.3389/fmicb.2021.601490 (PMC8032980; doi:10.3389/fmicb.2021.601490)
Supplement: Supplementary file 3 [file Table_3.DOCX]

Table 1: List of 7 *Enterococcus* virulence genes reported in the literature to be associated with human infections. Protein names and molecular functions are based on UniProt (<http://www.uniprot.org/>).

| **Gene name** | **Protein name** | **Protein function** | **Description** |
| --- | --- | --- | --- |
| *ace* | Collagen adhesin | Collagen binding | The *E. faecalis* specific gene ***ace*** encodes for a collagen-binding adhesin, which is shown to be important virulence factor associated with *E.* *faecalis* binding to mammalian cells as well as to collagens found in the extracellular matrix (Hall et al., 2007). |
| *asa1* | Aggregation substance | Peptidoglycan anchor | The ***asa1*** gene is carried by the sex pheromone plasmid pAD1 and encodes surface-bound glycoprotein that allows bacteria to form aggregates thereby facilitating conjugative plasmid transfer. As a virulence factor, Asa1 increases *Enterococcus* binding to immobilized fibronectin exposed on the injured epithelium and endothelium and is thereby associated with facilitating colonization and infection (Sartingen et al., 2000; Sussmuth et al., 2000; Rozdzinski et al., 2001). |
| *cylA* | Cytolysin CylA | Hydrolase, protease, serine protease | The ***cylA*** gene can be located in plasmids like pAD1 or in chromosome and it encodes activator protein of the extracellular toxin cytolysin, which is capable of lysing a wide-range of gram-positive bacteria as well as human erythrocytes (Ike and Clewell, 1992; Segarra, 1992). |
| *efaA_fs_* | *E. faecalis* endocarditis antigen A | Cell wall adhesin | The ***efaA_fs_*** gene encodes for the adhesin-like antigen, which is involved in *E. faecalis* adhesion to abiotic or biotic surfaces and plays role in the pathogenesis of endocarditis (Singh et al., 1998; Stępień-Pyśniak et al., 2019). |
| *efaA_fm_* | *E. faecium* endocarditis antigen A | Cell wall adhesin | The ***efaA_fm_*** gene encodes for the adhesin-like antigen that is expressed in serum by *E. faecium*. This gene is presumed to be involved in *E. faecium* adhesion to abiotic and biotic surfaces, although its exact role is yet unknown (Eaton and Gasson, 2001; Valenzuela et al., 2008; Soheili et al., 2014; Stępień-Pyśniak et al., 2019). |
| *espfs, espfm* | Surface protein Esp | - | The *E.faecium* gene ***espfm*** and *E.faecalis* gene ***espEs*** encode for a high-molecular-weight surface protein with unknown function. Nevertheless, it has been shown to be important in biofilm formation and thereby in infection pathogenesis (Toledo-Arana et al., 2001; Heikens et al., 2007; Freitas et al., 2018b). |
| *gelE* | Gelatinase | Hydrolase, metalloprotease, protease | The ***gelE*** encodes a secreted metalloprotease known as gelatinase or coccolysin, which can cleave gelatin, collagen, casein, hemoglobin as well as human vasoconstrictor endothelin-1 possibly related to inflammatory conditions during human infection (Mäkinen and Mäkinen, 1994; Waters et al., 2003; Alebouyeh et al., 2005). |
| *hylA* (*ef0323*)*,* *hylB* (*ef0818*) | Polysaccharide lyase, hyaluronate lyase, hyaluronidase | Lyase | The *E.* faecalis genes ***hylA*** and ***hylB*** encode a degradative enzyme hyaluronidase, which increases bacterial invasiveness by depolymerizing the mucopolysaccharides of connective tissues (Ørstavik, 2004). |
| *hylEfm* | Glycoside hydrolase | Hydrolase | The *E. faecium* gene ***hylEfm*** is carried by large mega-plasmid and encodes for a glycoside hydrolase, which has been considered as an important virulence factor associated with intestinal colonization and peritoneal invasion, although its role in virulence is yet undetermined (Rice et al., 2003; Freitas et al., 2010, 2018b; Panesso et al., 2011). |
| *acm* (*fms8*) | Collagen-binding MSCRAMM Acm (Fms8) | Collagen binding | The *E. faecium* gene ***acm*** encodes a collagen-binding adhesin, which intact form is shown to be necessary and sufficient for the *E. faecium* ability to bind to collagen type I (Nallapareddy et al., 2003; Freitas et al., 2018b). |
| *scm* (*fms10*) | Collagen-binding MSCRAMM Scm (Fms10), Second collagen adhesin | Collagen binding | The *E. faecium* gene ***scm*** encodes a adhesin with binding specificity to collagen types IV and V (Sillanpää et al., 2008; Freitas et al., 2018b). |
| *ecbA* (*orf2430*) | Collagen binding MSCRAMM EcbA | Collagen binding | The gene ***ecbA*** is in enriched in *E. faecium* clinical isolates and encodes a adhesin which binds to collagen type V and fibrinogen (Hendrickx et al., 2009; Freitas et al., 2018b). |
| *ptsD* | PTS system mannose/fructose family transporter subunit IID | Carbohydrate transport | The ***ptsD*** gene encodes a sugar-specific enzyme IID subunit of a membrane associated complex of a putative phosphotransferase system, which is involved in the active transport of carbohydrates over the cytoplasmatic membrane and contributes to intestinal colonization of clinical *E. faecium* strains (Zhang et al., 2013; Freitas et al., 2018a). |
| IS*16* | IS256-like element IS16 family transposase | Transposase | The **IS*16*** encodes a transposase, which facilitates the acquisition of mobile genetic elements. This transposase is enriched in clinical *E. faecium* strains and may support their spreading potential in nosocomial settings (Werner et al., 2011; Freitas et al., 2018a). |
| *orf1481* | - | Sugar-binding protein | The ***orf1481*** putatively encodes a sugar-binding protein and is located in a genomic island, which is involved in carbohydrate transport and metabolism and may provide clinical *E. faecium* isolates a competitive advantage in host colonization (Heikens et al., 2007; Freitas et al., 2018a). |
| *sgrA* (*orf2351*) | Serine-glutamate repeat containing protein A or | LPXTG-anchored fibrinogen/nidogen-binding adhesin | The ***sgrA*** gene encodes an adhesin, which is enriched in *E.* faecium clinical isolates. This adhesin binds to fibrinogen and basal lamina nidogens and is implicated in biofilm formation (Hendrickx et al., 2009; Freitas et al., 2018b). |
| pili gene cluster-1, *fms21* (*pilA*)-*fms20*, | Isopeptide-forming domain-containing fimbrial proteins Fms21 (PilA) and Fms20 | Major pilus subunit protein Fms21 and accessory pilus subunit protein Fms 20 | The ***fms21*** and ***fms20*** genes encode for *E. faecium* pili proteins and are located in a cluster, which is carried by a large plasmid shown to enhance *E. faecium’*s virulence and host’s gut colonization (Sillanpää et al., 2008; Kim et al., 2010; Freitas et al., 2018b). |
| pili gene cluster-2, *fms14*-*fms17*-*fms13* | VWA domain-containing protein Fms14, peptidase Fms17, SpaH/EbpB family LPXTG-anchored major pilin protein Fms13 | Major pilus subunit protein Fms13 and accessory pilus subunits Fms14 and Fms17 | The ***fms14***, ***fms17*** and ***fms20*** genes are located in a cluster and encode for *E. faecium* pili proteins (Sillanpää et al., 2008; Freitas et al., 2018b). |
| pili gene cluster-3, *ebpA_fm_* (*fms1*), *ebpB_fm_* (*fms5*), *ebpC_fm_* (*fms9, pilB*) | VWA domain-containing protein EbpA_fm_ (Fms1), peptidase EbpB_fm_ (Fms5), SpaH/EbpB family LPXTG-anchored major pilin protein EbpC_fm_ (Fms9, PilB) | Major pilus subunit protein EbpC_fm_ (Fms9, PilB) and accessory pilus subunits EbpA_fm_ (Fms1) and EbpB_fm_ (Fms5) | The ***ebpA_fm_***, ***ebpB_fm_*** and ***ebpC_fm_*** genes form a cluster, which is transcribed as an operon and encodes a pili on the *E. faecium* cell surface. This pili is enriched in *E. faecium* clinical isolates and is important for this bacteria’s ability to form biofilm and to cause infection (Sillanpää et al., 2008, 2010; Freitas et al., 2018b). |
| pili gene cluster-4, *fms11*-*fms19*-*fms16* | LPXTG family cell surface protein Fms11, collagen binding protein Fms19, SpaH/EbpB family LPXTG-anchored major pilin protein Fms16 | Major pilus subunit protein Fms16 and accessory pilus subunits Fms11 and Fms19 | The ***fms11***, ***fms19*** and ***fms16*** genes encode pili proteins and are located in a cluster, which is putatively associated with biofilm formation and is strongly enriched in ampicillin-resistant *E. faecium* strains (Sillanpää et al., 2008; Freitas et al., 2018b; Golob et al., 2019). |

# References

Alebouyeh, M., Amirmozafari, N., and Forohesh, H. (2005). Evaluation of virulence factors and plasmid-related transmissibility among different isolates of Enterococci. *Iran. Biomed. J.* 9, 51–55.

Eaton, T. J., and Gasson, M. J. (2001). Molecular Screening of Enterococcus Virulence Determinants and Potential for Genetic Exchange between Food and Medical Isolates. *Appl. Environ. Microbiol.* 67, 1628–1635. doi:10.1128/AEM.67.4.1628-1635.2001.

Freitas, A. R., Tedim, A. P., Novais, C., Coque, T. M., and Peixe, L. (2018a). Distribution of putative virulence markers in Enterococcus faecium: towards a safety profile review. *J. Antimicrob. Chemother.* 73, 306–319. doi:10.1093/jac/dkx387.

Freitas, A. R., Tedim, A. P., Novais, C., Coque, T. M., and Peixe, L. (2018b). Distribution of putative virulence markers in Enterococcus faecium: towards a safety profile review. *J. Antimicrob. Chemother.* 73, 306–319. doi:10.1093/jac/dkx387.

Freitas, A. R., Tedim, A. P., Novais, C., Ruiz-Garbajosa, P., Werner, G., Laverde-Gomez, J. A., et al. (2010). Global spread of the hylEfm colonization-virulence gene in megaplasmids of the Enterococcus faecium CC17 polyclonal subcluster. *Antimicrob. Agents Chemother.* 54, 2660–2665. doi:10.1128/AAC.00134-10.

Golob, M., Pate, M., Kušar, D., Dermota, U., Avberšek, J., Papić, B., et al. (2019). Antimicrobial Resistance and Virulence Genes in Enterococcus faecium and Enterococcus faecalis from Humans and Retail Red Meat. *Biomed Res. Int.* 2019. doi:10.1155/2019/2815279.

Hall, A. E., Gorovits, E. L., Syribeys, P. J., Domanski, P. J., Ames, B. R., Chang, C. Y., et al. (2007). Monoclonal antibodies recognizing the Enterococcus faecalis collagen-binding MSCRAMM Ace: Conditional expression and binding analysis. *Microb. Pathog.* 43, 55–66. doi:10.1016/j.micpath.2007.03.003.

Heikens, E., Bonten, M. J. M., and Willems, R. J. L. (2007). Enterococcal surface protein esp is important for biofilm formation of Enterococcus faecium E1162. *J. Bacteriol.* 189, 8233–8240. doi:10.1128/JB.01205-07.

Hendrickx, A. P. A., Van Luit-Asbroek, M., Schapendonk, C. M. E., Van Wamel, W. J. B., Braat, J. C., Wijnands, L. M., et al. (2009). SgrA, a nidogen-binding LPXTG surface adhesin implicated in biofilm formation, and EcbA, a collagen binding MSCRAMM, are two novel adhesins of hospital-acquired Enterococcus faecium. *Infect. Immun.* 77, 5097–5106. doi:10.1128/IAI.00275-09.

Ike, Y., and Clewell, D. B. (1992). Evidence that the hemolysin/bacteriocin phenotype of Enterococcus faecalis subsp. zymogenes can be determined by plasmids in different incompatibility groups as well as by the chromosome. *J. Bacteriol.* 174, 8172–8177. doi:10.1128/jb.174.24.8172-8177.1992.

Kim, D. S., Singh, K. V., Nallapareddy, S. R., Qin, X., Panesso, D., Arias, C. A., et al. (2010). The fms21 (pilA)-fms20 locus encoding one of four distinct pili of Enterococcus faecium is harboured on a large transferable plasmid associated with gut colonization and virulence. *J. Med. Microbiol.* 59, 505–507. doi:10.1099/jmm.0.016238-0.

Mäkinen, P. L., and Mäkinen, K. K. (1994). The enterococcus faecalis extracellular metalloendopeptidase (EC 3.4.24.30; coccolysin) inactivates human endothelin at bonds involving hydrophobic amino acid residues. *Biochem. Biophys. Res. Commun.* 200, 981–985. doi:10.1006/bbrc.1994.1546.

Nallapareddy, S. R., Weinstock, G. M., and Murray, B. E. (2003). Clinical isolates of Enterococcus faecium exhibit strain-specific collagen binding mediated by Acm, a new member of the MSCRAMM family. *Mol. Microbiol.* 47, 1733–1747. doi:10.1046/j.1365-2958.2003.03417.x.

Ørstavik, D. (2004). V Irulence F Actors of E Nterococcus Faecalis : 15, 308–320.

Panesso, D., Montealegre, M. C., Rincán, S., Mojica, M. F., Rice, L. B., Singh, K. V., et al. (2011). The hylEfm gene in pHylEfm of Enterococcus faecium is not required in pathogenesis of murine peritonitis. *BMC Microbiol.* 11, 20. doi:10.1186/1471-2180-11-20.

Rice, L. B., Carias, L., Rudin, S., Vael, C., Goossens, H., Konstabel, C., et al. (2003). A Potential Virulence Gene, *hyl* _Efm_ , Predominates in *Enterococcus faecium* of Clinical Origin. *J. Infect. Dis.* 187, 508–512. doi:10.1086/367711.

Rozdzinski, E., Marre, R., Susa, M., Wirth, R., and Muscholl-Silberhorn, A. (2001). Aggregation substance-mediated adherence of Enterococcus faecalis to immobilized extracellular matrix proteins. *Microb. Pathog.* 30, 211–220. doi:10.1006/mpat.2000.0429.

Sartingen, S., Rozdzinski, E., Muscholl-Silberhorn, A., and Marre, R. (2000). Aggregation substance increases adherence and internalization, but not translocation, of Enterococcus faecalis through different intestinal epithelial cells in vitro. *Infect. Immun.* 68, 6044–6047. doi:10.1128/IAI.68.10.6044-6047.2000.

Segarra, R. A. (1992). Molecular characterization of the Enterococcus faecalis hemolysin/bacteriocin determinant. 59, 1239–1246.

Sillanpää, J., Nallapareddy, S. R., Prakash, V. P., Qin, X., Höök, M., Weinstock, G. M., et al. (2008). Identification and phenotypic characterization of a second collagen adhesin, Scm, and genome-based identification and analysis of 13 other predicted MSCRAMMs, including four distinct pilus loci, in Enterococcus faecium. *Microbiology* 154, 3199–3211. doi:10.1099/mic.0.2008/017319-0.

Sillanpää, J., Nallapareddy, S. R., Singh, K. V., Prakash, V. P., Fothergill, T., Ton-That, H., et al. (2010). Characterization of the ebpfm pilus-encoding operon of enterococcus faecium and its role in biofilm formation and virulence in a murine model of urinary tract infection. *Virulence* 1, 236–246. doi:10.4161/viru.1.4.11966.

Singh, K. V, Coque, T. M., Weinstock, G. M., and Murray, B. E. (1998). In vivo testing of an *Enterococcus faecalis efaA* mutant and use of *efaA* homologs for species identification. *FEMS Immunol. Med. Microbiol.* 21, 323–331. doi:10.1111/j.1574-695X.1998.tb01180.x.

Soheili, S., Ghafourian, S., Sekawi, Z., Neela, V., Sadeghifard, N., Ramli, R., et al. (2014). Wide distribution of virulence genes among Enterococcus faecium and Enterococcus faecalis clinical isolates. *Sci. World J.* 2014. doi:10.1155/2014/623174.

Stępień-Pyśniak, D., Hauschild, T., Kosikowska, U., Dec, M., and Urban-Chmiel, R. (2019). Biofilm formation capacity and presence of virulence factors among commensal Enterococcus spp. from wild birds. *Sci. Rep.* 9, 1–7. doi:10.1038/s41598-019-47602-w.

Sussmuth, S. D., Muscholl-Silberhorn, A., Wirth, R., Susa, M., Marre, R., and Rozdzinski, E. (2000). Aggregation substance promotes adherence, phagocytosis, and intracellular survival of Enterococcus faecalis within human macrophages and suppresses respiratory burst. *Infect. Immun.* 68, 4900–4906. doi:10.1128/IAI.68.9.4900-4906.2000.

Toledo-Arana, A., Valle, J., Solano, C., Arrizubieta, M. J., Cucarella, C., Lamata, M., et al. (2001). The Enterococcal Surface Protein, Esp, Is Involved in Enterococcus faecalis Biofilm Formation. *Appl. Environ. Microbiol.* 67, 4538–4545. doi:10.1128/AEM.67.10.4538-4545.2001.

Valenzuela, A. S., Omar, N. Ben, Abriouel, H., López, R. L., Ortega, E., Cañamero, M. M., et al. (2008). Risk factors in enterococci isolated from foods in Morocco: Determination of antimicrobial resistance and incidence of virulence traits. *Food Chem. Toxicol.* 46, 2648–2652. doi:10.1016/j.fct.2008.04.021.

Waters, C. M., Antiporta, M. H., Murray, B. E., and Dunny, G. M. (2003). Role of the Enterococcus faecalis Ge1E protease in determination of cellular chain length, supernatant pheromone levels, and degradation of fibrin and misfolded surface proteins. *J. Bacteriol.* 185, 3613–3623. doi:10.1128/JB.185.12.3613-3623.2003.

Werner, G., Fleige, C., Geringer, U., van Schaik, W., Klare, I., and Witte, W. (2011). IS element IS16 as a molecular screening tool to identify hospital-associated strains of Enterococcus faecium. *BMC Infect. Dis.* 11, 80. doi:10.1186/1471-2334-11-80.

Zhang, X., Top, J., de Been, M., Bierschenk, D., Rogers, M., Leendertse, M., et al. (2013). Identification of a Genetic Determinant in Clinical Enterococcus faecium Strains That Contributes to Intestinal Colonization During Antibiotic Treatment. *J. Infect. Dis.* 207, 1780–1786. doi:10.1093/infdis/jit076.
