# Supplementary material for: Molecular Characterization of Enterococcus Isolates From Different Sources in Estonia Reveals Potential Transmission of Resistance Genes Among Different Reservoirs
Source: Front Microbiol. 2021 Mar 26;12:601490. doi: 10.3389/fmicb.2021.601490 (PMC8032980; doi:10.3389/fmicb.2021.601490)
Supplement: Supplementary file 4 [file Table_4.DOCX]

**Table 2:** List of the antimicrobial resistance genes found from our *Enterococcus* isolates. Protein names are based on UniProt (<http://www.uniprot.org/>).

| **Resistance gene** | **Protein name** | **Resistance to** | **Description** |
| --- | --- | --- | --- |
| *aac* | Aminoglycoside N-acetyltransferase | Aminoglycosides | The ***aac*** genes encode for the aminoglycoside *N-*acetyltransferases (AAC-s), which inactivate the aminoglycoside antibiotics by catalyzing the acetylation of –NH_2_ groups in the aminoglycoside molecules using acetyl coenzyme A as donor substrate (Ramirez and Tolmasky, 2010). *E. faecium* possess species-specific 6’-N-aminoglycoside acetyltransferase encoded by chromosomal *aac(6’)-Ii_1_L12710* gene. This intrinsic aminoglycoside *N-*acetyltransferase accounts for the loss of synergy between the substrate aminoglycoside and penicillins in this species. In both *E. faecalis* and *E. faecium,* the high-level resistance to aminoglycosides is conferred by bifunctional aminoglycoside phosphotransferase(2”)/ aminoglycoside acetyltransferase(6’) encoded by plasmid or transposon carried *aac(6’)-aph(2’’)_1_M13771* gene (Costa et al., 1993). |
| *ant, str_1, str_2* | Aminoglycoside O-nucleotidyltransferase | Aminoglycosides | The ***ant*** and ***str*** genes could be located in plasmids or in chromosomes and they encode for the aminoglycoside O-nucleotidyltransferases (ANT-s), which inactivate the aminoglycoside antibiotics by catalyzing the adenylylation of aminoglycoside molecules. Enzymes from classes ANT(6), ANT(4’) and ANT(9) have been described among *Enterococcus*. The ANT(6)-class enzymes inactivate the streptomycin molecules and are encoded by *ant(6)* and *str* genes found on both chromosomes and plasmids often as part of Tn*5405* transposones. The ANT(4’)-class enzymes can inactivate tobramycin, amikacin, isepamicin and dipecamicin molecules and are encoded by plasmid located *ant(4’)* genes. The ANT(9)-class tranferases inactivate the spectinomycin molecules and are encoded by *ant(9)* genes which are often found as part of Tn*554* transposone (Ramirez and Tolmasky, 2010). |
| *aph* | Aminoglycoside O-phosphatidyltransferase | Aminoglycosides | ***aph*** genes encode for aminoglycoside O-phosphotransferases (APH-s), which inactivate the aminoglycoside antibiotics by catalyzing the transfer of a phosphate group to the aminoglycoside molecule. The most prevalent aminoglycoside O-phosphotransferases are APH(3’)s of which APH(3’)-IIIa’s appears to be most prevalent among *Enterococcus*. APH(3’)-IIIa’s confer resistance to kanamycin, neomycin, lividomycin, paromomycin, livostamycin, butirosin, amikacin and isepamicin, but are completely inhibited by tobramycin (Badarau et al., 2008; Ramirez and Tolmasky, 2010; Woegerbauer et al., 2014; Khani et al., 2016). |
| *cat* | Chloramphenicol acetyltransferase | Chloramphenicol | ***cat*** genes encode for chloramphenicol acetyltransferases which inactivate the chloramphenicol antibiotics by catalyzing the acetylation of 3- or 1,3-hydroxyl groups using acetyl coenzyme A as donor substrate. Three most common chloramphenicol acetyltransferase encoding genes described in *Enterococcus* are cat(pC194), cat(pC221) and cat(pC223) carried by staphylococcal plasmids (Suzuki and Okamoto, 1967; Trieu-Cuot et al., 1993; Freitas et al., 2017). |
| *dfr* | Dihydrofolate reductase | Trimethoprim | ***dfr*** genes are mostly found in plasmids and encode for trimethoprim resistant dihydrofolate reductases (DHFRs). In addition to intrinsic *dfrE* gene, few transmissible DHFR genes have been described in *Enterococcus*, for example *dfrE dfrK* and *dfrG* (Coque et al., 1999; Sekiguchi et al., 2005; López et al., 2012; Vijayashree Priyadharsini et al., 2018)*.* |
| *erm* | Erythromycin resistance protein | Macrolide, lincosamide, streptogramin B | ***erm*** genes encode for the erythromycin resistance methylases, which methylate the adenine residue A2058 of 23S rRNA and thereby prevent the binding of macrolide, lincoasmide and streptogramin B (MLS_B_) antibiotics to target site (Szczuka et al., 2016). In *Enterococcus* *erm(A)*, *erm(B)* and *erm(C)* genes have been described in the plasmids and transposons, with the *erm(B)* being the most frequent and the *erm(C)* being the most infrequent cause of MLS_B_ resistance (Lim, 2002; Mikalsen et al., 2015; Tian et al., 2019). |
| *lsa* | ABC-F type ribosomal protection protein Lsa(A) | Lincosamide, pleuromutilins, streptogramin A | ***lsa*** genes encode for ABCF family ATPases which protect the ribosome peptidyl transferase center (PTC) by promoting the release of antibiotics like lincosamides, pleuromutilins and streptogramin A-s (Murina et al., 2019). In *Enterococcus* *lsa(A)* and *lsa(E)* genes have been described. *lsa(A)* is located in the core genome of *E. faecalis* and confers this species intrinsic resistance to lincosamides, pleuromutilins and streptogramin A. *lsa(E)* have been found in mobile genetic elements in both *E. faecalis* and *E. faecium* (Malbruny et al., 2011; Wendlandt et al., 2013). |
| *lnu* | Lincosamide nucleotidyltransferase | Lincosamide | ***lnu*** genes are usually located in plasmids or transposons and encode for the nucleotidyltransferases which specifically inactivate lincosamide antibiotics by catalyzing the O-adenylation of the hydroxyl group at the positions 3 or 4. In *Enterococc*us *lnu(B)*, *lnu(C)*, *lnu(D)* and *lnu(G)* genes have been described (Gravey et al., 2013; Zhu et al., 2017). |
| *msrC* | MsrC | Macrolide- streptogramin B | *E. faecium* harbors chromosomal ***msrC*** gene encoding for the ABCF family ATPase MsrC, which binds to the ribosome E-site and protects ribosome from the binding of macrolide and streptogramin B antibiotics (Murina et al., 2019). |
| *tetM, tetO* | Tetracyclin resistance protein TetM | Tetracyclin, minocyclin | ***tetM*** and ***tetO*** genes are located in plasmids or chromosomes and they encode for the ribosomal protection proteins which confer tetracycline resistance by catalyzing the release of tetracycline from ribosomes (Speer et al., 1992; Dönhöfer et al., 2012). |
| *tetL* | Tetracyclin resistance protein TetL | Tetracyclin, minocyclin | ***tetL*** genes are carried by plasmids and encode for cytoplasmic membrane transporter which confers tetracycline and minocycline resistance by active efflux of these antibiotics (McMurry et al., 1987; Speer et al., 1992). |
| *vanB* | Vancomycin B-type resistance protein VanB | Vancomycin | Genes in the ***vanB*** gene cluster (***vanH_B_****_,_* ***vanX_B_****,* ***vanY_B_****,* ***vanR_B_****,* ***vanS_B_***) account for producing altered from D-Ala-D-Lac peptidoglycane which has 1000-fold lower affinity to vancomycin than the normal D-Ala-D-Ala peptidoglycan (Lessard et al., 1998; Cetinkaya et al., 2000). |

# References

Badarau, A., Shi, Q., Chow, J. W., Zajicek, J., Mobashery, S., and Vakulenko, S. (2008). Aminoglycoside 2″-phosphotransferase type IIIa from Enterococcus. *J. Biol. Chem.* 283, 7638–7647. doi:10.1074/jbc.M709645200.

Cetinkaya, Y., Falk, P., and Mayhall, C. G. (2000). Vancomycin-resistant enterococci. *Clin. Microbiol. Rev.* 13, 686–707. doi:10.1128/CMR.13.4.686-707.2000.

Coque, T. M., Singh, K. V., Weinstock, G. M., and Murray, B. E. (1999). Characterization of dihydrofolate reductase genes from trimethoprim- susceptible and trimethoprim-resistant strains of Enterococcus faecalis. *Antimicrob. Agents Chemother.* 43, 141–147. doi:10.1128/aac.43.1.141.

Costa, Y., Galimand, M., Leclercq, R., Duval, J., and Courvalin, P. (1993). Characterization of the chromosomal aac(6’)-Ii gene specific for Enterococcus faecium. *Antimicrob. Agents Chemother.* 37, 1896–1903. doi:10.1128/AAC.37.9.1896.

Dönhöfer, A., Franckenberg, S., Wickles, S., Berninghausen, O., Beckmann, R., and Wilson, D. N. (2012). Structural basis for TetM-mediated tetracycline resistance. *Proc. Natl. Acad. Sci. U. S. A.* 109, 16900–16905. doi:10.1073/pnas.1208037109.

Freitas, A. R., Elghaieb, H., León-Sampedro, R., Abbassi, M. S., Novais, C., Coque, T. M., et al. (2017). Detection of optrA in the African continent (Tunisia) within a mosaic Enterococcus faecalis plasmid from urban wastewaters. *J. Antimicrob. Chemother.* 72, 3245–3251. doi:10.1093/jac/dkx321.

Gravey, F., Galopin, S., Grall, N., Auzou, M., Andremont, A., Leclercq, R., et al. (2013). Lincosamide resistance mediated by lnu(C) (L phenotype) in a Streptococcus anginosus clinical isolate. *J. Antimicrob. Chemother.* 68, 2464–2467. doi:10.1093/jac/dkt255.

Khani, M., Fatollahzade, M., Pajavand, H., Bakhtiari, S., and Abiri, R. (2016). Increasing prevalence of aminoglycoside-resistant enterococcus faecalis isolates due to the aac(6’)-aph(2’’”) Gene: A therapeutic problem in Kermanshah, Iran. *Jundishapur J. Microbiol.* 9. doi:10.5812/jjm.28923.

Lessard, I. A. D., Pratt, S. D., McCafferty, D. G., Bussiere, D. E., Hutchins, C., Wanner, B. L., et al. (1998). Homologs of the vancomycin resistance D-Ala-D-Ala dipeptidase VanX in Streptomyces toyocaensis, Escherichia coli and Synechocystis: Attributes of catalytic efficiency, stereoselectivity and regulation with implications for function. *Chem. Biol.* 5, 489–504. doi:10.1016/S1074-5521(98)90005-9.

Lim, J.-A. (2002). Prevalence of resistance to macrolide, lincosamide and streptogramin antibiotics in Gram-positive cocci isolated in a Korean hospital. *J. Antimicrob. Chemother.* 49, 489–495. doi:10.1093/jac/49.3.489.

López, M., Kadlec, K., Schwarz, S., and Torres, C. (2012). First detection of the staphylococcal trimethoprim resistance gene dfrK and the dfrK-carrying transposon Tn559 in enterococci. *Microb. Drug Resist.* 18, 13–18. doi:10.1089/mdr.2011.0073.

Malbruny, B., Werno, A. M., Murdoch, D. R., Leclercq, R., and Cattoir, V. (2011). Cross-resistance to lincosamides, streptogramins A, and pleuromutilins due to the lsa(C) gene in Streptococcus agalactiae UCN70 (Antimicrobial Agents and Chemotherapy (2011) 55, 4 (1470-1474)). *Antimicrob. Agents Chemother.* 55, 3065. doi:10.1128/AAC.00444-11.

McMurry, L. M., Park, B. H., Burdett, V., and Levy, S. B. (1987). Energy-dependent efflux mediated by class L (TetL) tetracycline resistance determinant from streptococci. *Antimicrob. Agents Chemother.* 31, 1648–1650. doi:10.1128/AAC.31.10.1648.

Mikalsen, T., Pedersen, T., Willems, R., Coque, T. M., Werner, G., Sadowy, E., et al. (2015). Investigating the mobilome in clinically important lineages of enterococcus faecium and enterococcus faecalis. *BMC Genomics* 16, 1–16. doi:10.1186/s12864-015-1407-6.

Murina, V., Kasari, M., Takada, H., Hinnu, M., Saha, C. K., Grimshaw, J. W., et al. (2019). ABCF ATPases Involved in Protein Synthesis, Ribosome Assembly and Antibiotic Resistance: Structural and Functional Diversification across the Tree of Life. *J. Mol. Biol.* 431, 3568–3590. doi:10.1016/j.jmb.2018.12.013.

Ramirez, M. S., and Tolmasky, M. E. (2010). Aminoglycoside modifying enzymes. *Drug Resist. Updat.* 13, 151–171. doi:10.1016/j.drup.2010.08.003.

Sekiguchi, J. I., Tharavichitkul, P., Miyoshi-Akiyama, T., Chupia, V., Fujino, T., Araake, M., et al. (2005). Cloning and characterization of a novel trimethoprim-resistant dihydrofolate reductase from a nosocomial isolate of Staphylococcus aureus CM.S2 (IMCJ1454). *Antimicrob. Agents Chemother.* 49, 3948–3951. doi:10.1128/AAC.49.9.3948-3951.2005.

Speer, B. S., Shoemaker, N. B., and Salyers, A. A. (1992). Bacterial resistance to tetracycline: Mechanisms, transfer, and clinical significance. *Clin. Microbiol. Rev.* 5, 387–399. doi:10.1128/CMR.5.4.387.

Suzuki, Y., and Okamoto, S. (1967). The enzymatic acetylation of chloramphenicol by the multiple drug-resistant Escherichia coli carrying R factor. *J. Biol. Chem.* 242, 4722–4730.

Szczuka, E., Makowska, N., Bosacka, K., Słotwińska, A., and Kaznowski, A. (2016). Molecular basis of resistance to macrolides, lincosamides and streptogramins in Staphylococcus hominis strains isolated from clinical specimens. *Folia Microbiol. (Praha).* 61, 143–147. doi:10.1007/s12223-015-0419-6.

Tian, Y., Yu, H., and Wang, Z. (2019). Distribution of acquired antibiotic resistance genes among Enterococcus spp. isolated from a hospital in Baotou, China. *BMC Res. Notes* 12, 12–16. doi:10.1186/s13104-019-4064-z.

Trieu-Cuot, P., De Cespedes, G., Bentorcha, F., Delbos, F., Gaspar, E., and Horaud, T. (1993). Study of heterogeneity of chloramphenicol acetyltransferase (CAT) genes in streptococci and enterococci by polymerase chain reaction: Characterization of a new CAT determinant. *Antimicrob. Agents Chemother.* 37, 2593–2598. doi:10.1128/AAC.37.12.2593.

Vijayashree Priyadharsini, J., Smiline Girija, A. S., and Paramasivam, A. (2018). Enterococcus faecalis an emerging microbial menace in dentistry-An insight into the in-silico detection of drug resistant genes and its protein diversity. *J. Clin. Diagnostic Res.* 12, GC06-GC10. doi:10.7860/JCDR/2018/36480.12155.

Wendlandt, S., Lozano, C., Kadlec, K., Gómez-Sanz, E., Zarazaga, M., Torres, C., et al. (2013). The enterococcal ABC transporter gene lsa(E) confers combined resistance to lincosamides, pleuromutilins and streptogramin A antibiotics in methicillin-susceptible and methicillinresistant Staphylococcus aureus. *J. Antimicrob. Chemother.* 68, 473–475. doi:10.1093/jac/dks398.

Woegerbauer, M., Zeinzinger, J., Springer, B., Hufnagl, P., Indra, A., Korschineck, I., et al. (2014). Prevalence of the aminoglycoside phosphotransferase genes aph(39)-IIIa and aph(39)-IIa in Escherichia coli, Enterococcus faecalis, Enterococcus faecium, Pseudomonas aeruginosa, Salmonella enterica subsp. Enterica and Staphylococcus aureus isolates in Aust. *J. Med. Microbiol.* 63, 210–217. doi:10.1099/jmm.0.065789-0.

Zhu, X. Q., Wang, X. M., Li, H., Shang, Y. H., Pan, Y. S., Wu, C. M., et al. (2017). Novel lnu(G) gene conferring resistance to lincomycin by nucleotidylation, located on Tn6260 from Enterococcus faecalis E531. *J. Antimicrob. Chemother.* 72, 993–997. doi:10.1093/jac/dkw549.
